# Variants of *GRM7* as risk factor and response to antipsychotic therapy in schizophrenia

**DOI:** 10.1038/s41398-020-0763-4

**Published:** 2020-03-03

**Authors:** Wei Liang, Hao Yu, Yi Su, Tianlan Lu, Hao Yan, Weihua Yue, Dai Zhang

**Affiliations:** 1 Institute of Mental Health, Peking University Sixth Hospital, 100191 Beijing, China; 2 NHC Key Laboratory of Mental Health, National Clinical Research Center for Mental Disorders and Key Laboratory of Mental Health, Ministry of Health (Peking University Sixth Hospital), 100191 Beijing, China; 3 Shandong Collaborative Innovation Center for Diagnosis, Treatment and Behavioral Interventions of Mental Disorders, Department of Psychiatry, Jining Medical University, 272067 Jining, Shandong China; 4 PKU-IDG/McGovern Institute for Brain Research, Peking University, 100871 Beijing, China; 5 Chinese Academy of Medical Sciences Research Unit (No. 2018RU006), Peking University, 100191 Beijing, China

**Keywords:** Pharmacogenetics, Clinical genetics

## Abstract

Genome-wide association study (GWAS) has determined the metabotropic glutamate receptor 7 (*GRM7*) gene as potential locus for schizophrenia risk variants; However, the relationship between the *GRM7* variants and the risk of schizophrenia is still uncertain, and there are significant individual variations in response to the antipsychotic drugs. In order to identify susceptible gene and drug-response-related markers, 2413 subjects in our research were chosen for determining drug-response-related markers in schizophrenia. The rs1516569 variant (OR = 0.95, *P* < 3.47 × 10^−4^) was a significant risk factor, and a single-nucleotide polymorphism of *GRM7* gene- rs9883258 (OR = 0.84, *P* = 2.18 × 10^−3^) has been determined as potential biomarkers for therapeutic responses of seven commonly used antipsychotic drugs (aripiprazole, haloperidol, olanzapine, perphenazine, quetiapine, risperidone and ziprasidone) in Chinese Han population; Significant associations with treatment response for several single-nucleotide polymorphisms in every antipsychotic drugs, such as rs779746 (OR = 1.39, *P* = 0.03), rs480409 (OR = 0.73, *P* = 0.04), rs78137319 (OR = 3.09, *P* = 0.04), rs1154370 (OR = 1.51, *P* = 0.006) have been identified in our study. Hence our research elucidates that *GRM7* variants play the critical role of predicting the risk of schizophrenia and antipsychotic effect of seven common drugs.

## Introduction

Schizophrenia (SCZ) is a debilitating psychiatric syndrome with decreased quality of life and shortened life expectancy, which affects around 1% of people at some point in their life^1,2^. Although the etiology of SCZ is still unknown, twin investigations have elucidated that it is highly inheritable neuropsychiatry disorder^3^. Probing into genetic mechanisms of SCZ has appealed to more and more concern.

Recently, SZDB (www.szdb.org) have identified hundreds of loci displaying a genome-wide significant correlation with SCZ in diverse population^4^. Of these risk loci, some are related major hypotheses of the etiology and treatment of SCZ, such as genes involved in glutamatergic (*GRM3*, *GRIN2A*, *GRIA1*) neurotransmission^5^. However, the roles of other glutamatergic genes in SCZ was still unclear.

One of the major hypotheses of SCZ is the glutamatergic neuronal dysfunction hypothesis^6^. Some single nucleotide polymorphisms (SNPs) of metabotropic glutamate receptor 7 (*GRM7*) have also been found to be related to SCZ in different populations^7–9^. *GRM7* is considered to be a critical actor in regulating synaptic glutamate transmission and γ-amino butyric acid (GABA) release in the central nervous system^9–11^. Whereas, though previous investigations have strongly revealed *GRM7* as the SCZ risk factor, these studies only focus on a small amount of SNPs in limited sample. To expand the knowledge of *GRM7* in SCZ susceptibility, researches with large sample data and with broad analysis are needed.

On the other hand, evidence implies that genetic variants of *GRM7* play a critical part in different antipsychotic drug response^12–14^. Nowadays, the Pharmacotherapy is the main way to improve the symptoms of schizophrenia^15^. According to the pharmacological research, the subjects have different personalized response to the seven common anti-antipsychotic drugs.It is necessary to determine the drug-efficient markers in order to predict the curative effect. Genetic predictors discovered by pharmacogenetic investigation could provide a clue for clinicians to select the reasonable therapy for SCZ patients. The previous researches have illustrated positive associations between genetic variation and efficacy of antipsychotic drugs treatment^16,17^. However, these studies mainly center at the European populations or include small samples. Therefore, these results need to be confirmed in diffident population and validated in concrete clinical application.

In this investigation, we employed GWAS samples from diverse populations to analyze a high density of SNPs in *GRM7* gene. The discovered SCZ risk SNPs were then detected for their correlation with *GRM7* expression. We hypothesized that *GRM7* might contribute to the treatment outcomes of antipsychotic drugs; therefore, we examined the associations between the *GRM7* genotype and treatment response of antipsychotic drug.

## Materials and methods

### SCZ GWAS data

We meta-analyzed *GRM7* associations across three GWAS datasets in diverse populations, via a fixed-effects model with inverse-variance weighting. The GWAS datasets were described as follows. Concrete information concerning sample description, diagnosis, genotyping and statistical analyses can be discovered in the original research^18–20^.

Asian sample: we used the GWAS data from the Chinese Schizophrenia Collaboration Group (CSCG)^19^ and Asian project of Psychiatric Genomics Consortium (PGC-Asian)^20^. The SCZ GWAS of CSCG is a meta-analysis of four independent schizophrenia GWAS datasets (GWAS1-GWAS4) were conducted, including a total of 4565 patients and 5947 controls^19^. All Han Chinese samples included were acquired from plentiful cooperating hospitals in the CSCG. Genotyping of the samples was conducted using Illumina (San Diego, CA, USA). Meanwhile, the PGC-Asian GWAS compiled 22,778 SCZ patients and 35,362 controls from 20 samples in Singapore, Japan, Indonesia, Korea, Hong Kong, Taiwan, and mainland China^20^. Meta-analyses across all PGC-Asian samples were conducted using a fixed-effects model with inverse-variance weighting. After removing sample overlap between CSCG and PGC-Asian (e.g., GWAS3 and GWAS4 of CSCG), 25,119 SCZ patients and 38,408 controls were used for the analysis.

European sample: The SCZ GWAS of European sample was from a large-scale SCZ GWAS of the PGC and CLOZUK study^18^. After excluding related and overlapping samples of PGC and CLOZUK study, it comprised of 40,675 cases and 64,643 controls. The SNP associations of *GRM7* gene were download from the website (http://walters.psycm.cf.ac.uk).

We performed a meta-analysis of the SNPs covering the *GRM7* region in all samples with PLINK v1.90, and used odds ratio (OR) and standard error (SE) for the estimation of heterogeneity between individual samples. The meta-analysis was performed using the classical inverse variance weighted methods. The regional association results of SNPs were plotted using LocusZoom^21^. After meta-analysis, we performed Linkage disequilibrium (LD)-based clumping with a SNP window of 500 kb and *r*^2^ = 0.1.

### Pharmacogenetics study of *GRM7* gene

#### Participants

To examine the associations between *GRM7* variations and treatment response in patients with SCZ, we performed a pharmacogenetics study using our previous data^22^. Briefly, all subjects were of Chinese Han ethnicity, and were born and residing in China. The consensus diagnoses were made by at least two experienced senior psychiatrists according to the Diagnosis and Statistic Manual of Mental Disorders, 4th edition (DSM-IV) criteria for schizophrenia. Patients with previously diagnosed diabetes, thyroid disease, hypertension, heart disease and other severe physical diseases were excluded. Patients who met inclusion criteria were randomly assigned (1:1:1:1:1:1:1) to seven groups (olanzapine, risperidone, quetiapine, aripiprazole, ziprasidone, and haloperidol or perphenazine) for six-week treatment.

Clinical effect was assessed based on the Positive and Negative Syndrome Scale (PANSS), which include the positive, negative and general psychopathology subscales. The patients visited the participating clinicians at weeks 2, 4, and 6, and their PANSS scores were then recorded. For each patient, the PANSS reduction rate was defined as 100× (PANSS score at endpoint-PANSS score at baseline)/(PANSS score at baseline -30). Different from our previous study^22^, the antipsychotic response was classed into two groups, clinical good responders were defined as patients with 50% or even higher reduction in PANSS scores; poor responders were defined as patients with lower reduction than 50% in PANSS scores. A detailed description of the participants can be found in our previous study^22^. The study was approved by the research ethics committees and institutional review boards of each local hospital. All procedures were conducted in accordance with the principles expressed in the Declaration of Helsinki. The study was registered under the clinical trial number ChiCTR-TRC-10000934 (http://www.chictr.org/).

#### Genotyping

Genomic DNA was extracted using the QIAamp DNA Mini Kit (QIAGEN, Hilden, Germany). Sample genotype sequencing was genotyped with Illumina Human Omni ZhongHua-8 Beadchips (Illumina, San Diego, CA, USA). Genotype imputation for the discovery sample. Genotype imputation was done with the pre-phasing imputation stepwise approach implemented in IMPUTE2 and SHAPEIT (version 2.r727)^17,21^. Haplotypes derived from phase I of the 1000 Genomes Project (release version 3) were used as references. SNPs with imputation quality scores below a set threshold (info score < 0.9) were excluded from further analyses. All genomic locations are given as National Center for Biotechnology Information Build 37 coordinates. We extracted the *GRM7* genotype from the imputed data. Totally, 652 SNPs in *GRM7* were extracted for this study. After LD-based pruning (a window of 500 SNPs and *r*^2^ = 0.1), 19 SNPs were retained for the pharmacogenetics study.

#### Statistical analysis

Cohort characteristics including gender, age and weight differences of demographic and clinical variables were examined first to affirm the homogeneity of good responders and poor responders in our samples. We used logistic regression under an additive genetic model to evaluate the associations between the allele dosages and the PANSS percentage change values in PLINK v1.90^23^. The *P* values were adjusted by Bonferroni correction.

### Gene expression analysis

We examined *cis*-eQTL data of *GRM7* gene in the Brain Expression Consortium (BRAINEAC, http://caprica.genetics.kcl.ac.uk/BRAINEAC/), which consisted of 134 neuropathologically normal donors from the MRC Sudden Death Brain Bank in Edinburgh and Sun Health Research Institute, the gene expression was profiled on the Affymetrix Exon 1.0 ST array^24^. In BRAINEAC database, we can examine generated eQTL data for ten human brain regions (cerebellar cortex, frontal cortex, hippocampus, inferior olivary nucleus, occipital cortex, putamen, substantia nigra, temporal cortex, thalamus and intralobular white matter)^24^. To explore whether the *GRM7* gene is differentially expressed in SCZ patients compared to controls, we obtained publicly available expression data from BrainSeq database^25^. The BrainSeq project performed RNA-seq analysis using DLPFC of 412 subjects (175 patients with SCZ and 237 unaffected controls)^25^.

## Results

### *GRM7* and the risk of SCZ

**Fig. 1:**
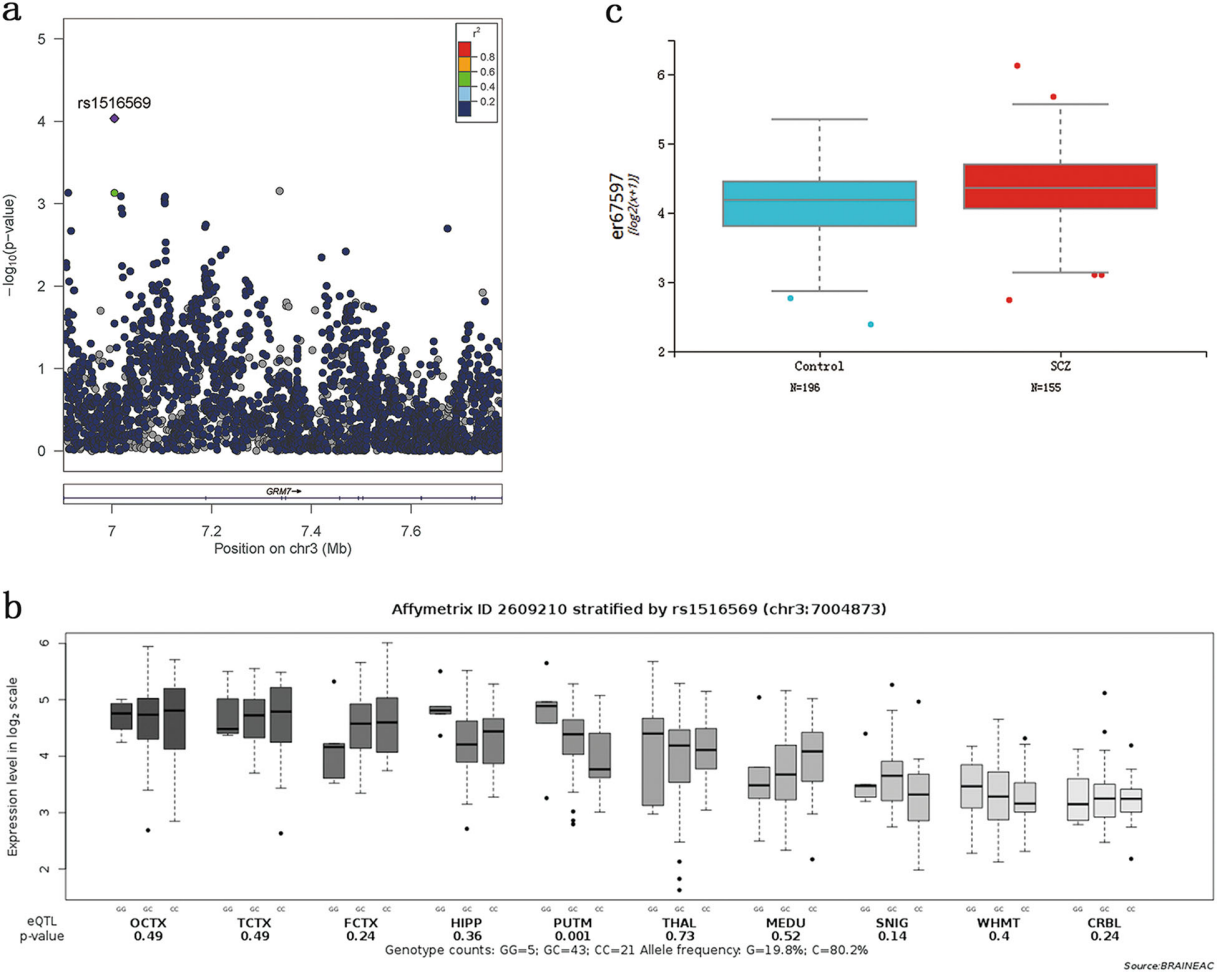
Association between the SNP rs1516569 of GRM7 and SCZ risk. **a** Genetic association of GRM7 SNPs with risk for SCZ in meta-analysis of three ethnic samples; **b**) eQTL analysis of the SNP rs1516569 in BRAINEAC database (http://caprica.genetics.kcl.ac.uk/BRAINEAC/)^24^; **c**) GRM7 gene is differentially expressed in SCZ patients compared to controls in BrainSeq databse (http://eqtl.brainseq.org/)^25^.

Demographic and descriptive data are listed in Table 1. The association data of 3273 SNPs spanning the *GRM7* region were acquired from both European and Asian GWAS samples, consisted of 65,794 cases and 103,051 controls. Then, a meta-analysis of these SNPs including all samples (Fig. 1a) was carried out. After LD-based clumping, 79 independent SNPs were retained. Only the SNP rs1516569 (OR = 0.95, *P* < 3.47 × 10^−4^, Table 2) was significantly associated with SCZ after Bonferroni correction (*P* < 0.05/79 = 6.30 × 10^−4^). However, the SNP rs1516569 is still far from genome-wide significance (*P* < 5 × 10^−8^). To examine whether rs1516569 also modulates the transcription and expression of *GRM7*, we employed the gene expression data in ten brain regions obtained from the BRAINEAC database. Interestingly, the risk allele (G) of rs1516569 (*P* = 1.0 × 10^−3^) was significantly correlated with higher *GRM7* expression in putamen region (Fig. 1b). Furthermore, we performed differential expression analysis of *GRM7* gene using BrainSeq database^25^. Compared with controls, the expression level of *GRM7* transcript was significantly higher in SCZ patients (*t* = 3.19, *P* = 1.55 × 10^−3^; Fig. 1c). These results suggested that the risk SNP probably was conducive to the disease risk by modulating *GRM7* expression of brain.

**Table 1:** Descriptive statistics for subjects in meta-analysis.

Analyses	Cases			Controls		
	Sample size	Mean age (s.d.)	Male/female	Sample size	Mean age (s.d.)	Male/female
GWAS1	746	34.5 (8.7)	396/350	1599	35.8 (7.8)	846/753
GWAS2	1595	30.1 (10.7)	859/736	1447	29.7 (9.8)	350/1090

*s.d.* standard deviation.

**Table 2:** The associations between the SNP rs1516569 of *GRM7* and schizophrenia.

Study	Chr	BP	SNP	A1	A2	OR	P
GWAS1 of CSCG^19^ (746 cases and 1599 controls)	3	7004873	rs1516569	C	G	1.06	0.66
GWAS2 of CSCG^19^ (1595 cases and 1447 controls)	3	7004873	rs1516569	C	G	1.11	0.12
PGC-Asian^20^ (22,778 cases and 35,362 controls)	3	7004873	rs1516569	C	G	0.97	0.72
PGC-Clozuk^18^ (40,675 cases and 64,643 controls)	3	7004873	rs1516569	C	G	0.95	7.63E-05
Meta-analysis 65,794 cases and 103,051 controls	3	7004873	rs1516569	C	G	0.85	3.47E-04

GWAS1 and GWAS2 of CSCG were not included in PGC Asian project. Therefore, we performed a meta-analysis of four GWAS datasets.

*CHR* chromosome, *SNP* single nucleotide polymorphism, *BP* base position, *A1/A2* minor allele/major allele, *OR* odds ratio.

### *GRM7* and treatment response of antipsychotic drugs

Demographic and descriptive data are listed in Table 3. Based on the assumption that genetic moderators of response are similar across antipsychotics, we first performed a combined analysis across seven antipsychotic drugs. Among 19 LD-independent SNPs, the SNP rs9883258 showed vital correlation with treatment responses to antipsychotic drugs (OR = 0.84, *P* = 2.18 × 10^−3^; Table 4). The result remains significant after Bonferroni correction (*P* < 2.63 × 10^−3^, i.e., 0.05/19).

**Table 3:** Descriptive statistics for antipsychotic patient-related variables.

Variable	Poor responders	Good responders
No.	991	1422
Male, No. (%)	498 (50.3)	738 (51.9)
Female, No. (%)	493 (49.7)	684 (48.1)
Age, mean (SD), y	31.4 (8.0)	31.5 (8.0)
Antipsychotic drugs		
Olanzapine	142	283
Risperidone	133	279
Aripiprazole	185	212
Quetiapine	172	214
Ziprasidone	199	209
Haloperidol	79	113
Perphenazine	81	112
PANSS total score, mean (SD)		
Baseline	11.8 (14.6)	12.5 (16.0)
6 week	18.3 (27.0)	9.8 (13.9)

**Table 4:** Genotype and allele frequencies of significant SNPs associated with antipsychotic treatment.

CHR	SNP	BP	A1	A2	F_A	F_U	OR	SE	L95	U95	P
3	rs9883258	7419156	G	A	0.4708	0.5156	0.84	0.06	0.75	0.94	0.00218
3	rs9882058	6937327	C	T	0.0116	0.0182	0.63	0.24	0.39	1.02	0.0578
3	rs7374553	7528503	G	A	0.4877	0.4617	1.11	0.06	0.99	1.25	0.0748
3	rs1154370	7062465	G	T	0.4863	0.4627	1.10	0.06	0.98	1.23	0.106
3	rs779746	7578531	C	T	0.4898	0.4662	1.10	0.06	0.98	1.23	0.1063
3	rs670764	7026341	G	A	0.4318	0.4092	1.10	0.06	0.98	1.23	0.1178
3	rs342026	6908535	G	A	0.4909	0.5111	0.92	0.06	0.82	1.03	0.1665
3	rs712767	7443688	C	T	0.4655	0.4521	1.06	0.06	0.94	1.19	0.3556
3	rs1504047	7748843	T	G	0.2996	0.3118	0.94	0.06	0.83	1.07	0.3639
3	rs13321431	7001961	T	G	0.4842	0.495	0.96	0.06	0.85	1.07	0.4612
3	rs480409	7010081	C	T	0.3952	0.3905	1.02	0.06	0.91	1.15	0.7421
3	rs78137319	7348332	A	G	0.01371	0.01261	1.09	0.26	0.66	1.80	0.7425
3	rs11918634	7683434	A	G	0.4205	0.4162	1.02	0.06	0.91	1.14	0.7664
3	rs74668910	7068619	T	G	0.01055	0.01009	1.05	0.29	0.59	1.85	0.8772
3	rs2291867	7340164	G	A	0.4965	0.4985	0.99	0.06	0.88	1.11	0.8911
3	rs17047886	7780116	C	T	0.01372	0.01413	0.97	0.25	0.60	1.58	0.906
3	rs6778030	7153652	G	A	0.4662	0.4647	1.01	0.06	0.90	1.13	0.9147
3	rs7623046	7510856	A	G	0.4989	0.5	1.00	0.06	0.89	1.12	0.9425
3	rs3749380	6903297	T	C	0.2827	0.2825	1.00	0.06	0.88	1.14	0.9905

*CHR* chromosome, *SNP* single nucleotide polymorphism, *BP* base position, *A1/A2* minor allele/major allele, *F_P* frequency of minor allele in patients of poor response, *F_G* frequency of minor allele in patients of good response, *L95/U95* 95% Confidence interval, *OR* odds ratio, *SE* standard error.

Then, we performed a second investigation in patients treated with specific antipsychotic drugs. Several SNPs were found to be nominally with several specific antipsychotic drug (Supplementary Tables 1–7). In olanzapine group, the SNPs rs779746 (OR = 1.39, *P* = 0.03; Supplementary Table 1) and rs480409 (OR = 0.73, *P* = 0.04; Supplementary Table 1) were nominally associated with treatment response. The SNP rs78137319 (OR = 3.09, *P* = 0.04; Supplementary Table 2) was nominally associated with treatment response of quetiapine. The SNP rs1154370 was nominally associated with treatment response of risperidone (OR = 1.51, *P* = 0.006; Supplementary Table 3) and haloperidol (OR = 1.73, *P* = 9.0 × 10^−3^; Supplementary Table 4). The SNP rs779746 (OR = 0.61, *P* = 0.02; Supplementary Table 5) was nominally associated with treatment response of perphenazine.

## Discussion

With regard to the core risk factors, the *GRM7* gene is significantly correlated with the schizophrenia patients. Glutamatergic neurotransmission may participate in many respects of normal brain activity and be disturbed in the state of neuropathology. The metabotropic glutamate receptors include three different groups and *GRM4, GRM6, GRM7*, and *GRM8* belong to Group III, Group II and III receptors play a role in inhibiting the cyclic AMP cascade^26^. The gene of *GRM7* include several transcript variants encoding various isoforms. Our meta-analysis showed that rs1516569 in *GRM7* was significantly associated with SCZ; this favorable SNP has not been determined in two earlier Caucasian independent samples^27,28^ and in the two Chinese samples^29,30^. The 1000 Genomes Project Phase 3 database (http://grch37.ensembl.org) showed that there are significant differences in the minor allele frequency (MAF) of rs1516569 across populations, especially between European (EUR) and East Asian (EAS) populations (Supplementary Fig. 1). The different SNPs between our study and the others may be derived from the sample size. Also a study shows that *GRM7* modulates the phosphorylation of cyclic AMP response element-binding protein (CREB) and the expression of Yes-associated protein (YAP) via direct interaction with CaM, which furtherly adjusts the expression of CyclinD1 and finally influences early cortical development^31^. And the factors affecting early development contribute to the etiology of schizophrenia. In summary, this study furtherly proved *GRM7* as the risk factor of schizophrenia.

In terms of antipsychotic drug-efficient pharmacogenetics studies, we found the limited sample recruitment in these studies. The small-sample-set may explain for the inconsistent results of researches on different groups. In our study, nearly 2413 patients were included for exploring the predictive markers. For the sake of determining the perplexing genetic factors involved in the efficacy of antipsychotic treatment, SNPs of *GRM7* gene participated in synaptic transmission and plasticity^32^ have been explored for seven common antipsychotic drugs, which uncover the mechanism of antipsychotic response concerning the specified drugs. In general, our study is a correlation analysis between genetic variants and the efficacy of olanzapine, risperidone, quetiapine, aripiprazole, ziprasidone, and haloperidol or perphenazine in the Chinese Schizophrenia.

Our research indicated that rs9883258 in *GRM7* was correlated with antipsychotic response which is an intron variant and overlaps 10 transcripts. The 1000 Genomes Project Phase 3 database (http://grch37.ensembl.org) showed that there are significant differences in the minor allele frequency (MAF) of rs9883258 across populations, especially between European (EUR) and East Asian (EAS) populations (Supplementary Fig. 2). The rs779746, rs480409 in *GRM7* were associated with olanzapine response, the rs779746 is an intron variant and overlaps 12 transcripts, the rs480409 overlaps 11 transcripts which was in line with the previous CATIE trial^33^; The rs78137319 was associated with treatment response of quetiapine which is a synonymous variant and overlaps 11 transcripts. The SNP rs1154370 was associated with treatment response of risperidone and haloperidol which is an intron variant overlaps 11 transcripts. The SNP rs779746 was associated with treatment response of perphenazine which is an intron variant and overlaps 12 transcripts. However, there are different *GRM7* SNPs in antipsychotic response for Schizophrenia patients. the investigation illustrated that rs2133450 in *GRM7* was significantly involved in Risperidone response^13^; The association between rs2069062, rs1532544 in *GRM7* and the response to lurasidone was determined in the research concerning European and African Ancestries^34^.

At the same time, this research resides in some limitations. Firstly, we did not include the patients who take the other antipsychotic treatment besides the seven drugs, which can narrow the implication of study. Secondly, the samples recruited were the Han Chinese, which will have relatively narrowed significance. Thirdly, we make an associated analysis by using logistic regression under an additive genetic model with our current data, furtherly more comprehensive antipsychotic sample sizes and predictive model may be helpful for more sound results. Although, there are some limitations of the study, our findings that *GRM7* was related to antipsychotic treatment efficacy could shed light on interactions between the *GRM7* and antipsychotics, which can provide a sound foundation for SCZ therapy.

## Supplementary information

Supplementary information
