# Supplementary material for: Variants of GRM7 as risk factor and response to antipsychotic therapy in schizophrenia
Source: Transl Psychiatry. 2020 Mar 3;10:83. doi: 10.1038/s41398-020-0763-4 (PMC7054263; doi:10.1038/s41398-020-0763-4)

**Supplementary Table 1. Association results of GRM7 SNPs with olanzapine treatment response**

| CHR | SNP | BP | A1 | A2 | F_P | F_G | OR | SE | L95 | U95 | P |
| --- | --- | --- | --- | --- | --- | --- | --- | --- | --- | --- | --- |
| 3 | rs779746 | 7578531 | C | T | 0.5071 | 0.4261 | 1.386 | 0.1465 | 1.04 | 1.847 | 0.02576 |
| 3 | rs480409 | 7010081 | C | T | 0.3728 | 0.4472 | 0.7348 | 0.1476 | 0.5501 | 0.9814 | 0.03659 |
| 3 | rs7623046 | 7510856 | G | A | 0.4664 | 0.5317 | 0.77 | 0.1457 | 0.5787 | 1.025 | 0.0726 |
| 3 | rs712767 | 7443688 | C | T | 0.4611 | 0.4014 | 1.276 | 0.1475 | 0.9557 | 1.704 | 0.09812 |
| 3 | rs6778030 | 7153652 | G | A | 0.4152 | 0.4683 | 0.8061 | 0.1463 | 0.6051 | 1.074 | 0.1404 |
| 3 | rs1504047 | 7748843 | T | G | 0.3039 | 0.3415 | 0.8416 | 0.1549 | 0.6212 | 1.14 | 0.2655 |
| 3 | rs78137319 | 7348332 | A | G | 0.0053 | 0.01056 | 0.4991 | 0.8198 | 0.1001 | 2.489 | 0.3873 |
| 3 | rs2291867 | 7340164 | G | A | 0.4629 | 0.493 | 0.8865 | 0.1456 | 0.6664 | 1.179 | 0.4077 |
| 3 | rs3749380 | 6903297 | T | C | 0.2862 | 0.3134 | 0.8786 | 0.1582 | 0.6444 | 1.198 | 0.4129 |
| 3 | rs1154370 | 7062465 | G | T | 0.4682 | 0.4965 | 0.8929 | 0.1455 | 0.6713 | 1.188 | 0.4362 |
| 3 | rs74668910 | 7068619 | T | G | 0.008834 | 0.01408 | 0.6239 | 0.6748 | 0.1662 | 2.342 | 0.4805 |
| 3 | rs670764 | 7026341 | G | A | 0.4258 | 0.4014 | 1.106 | 0.1479 | 0.8275 | 1.478 | 0.4965 |
| 3 | rs9882058 | 6937327 | C | T | 0.01237 | 0.01773 | 0.6937 | 0.5901 | 0.2182 | 2.206 | 0.5333 |
| 3 | rs7374553 | 7528503 | G | A | 0.4982 | 0.4789 | 1.081 | 0.1455 | 0.8124 | 1.437 | 0.5944 |
| 3 | rs13321431 | 7001961 | T | G | 0.4859 | 0.4683 | 1.073 | 0.1457 | 0.8065 | 1.427 | 0.6289 |
| 3 | rs17047886 | 7780116 | C | T | 0.01413 | 0.01056 | 1.343 | 0.6809 | 0.3535 | 5.101 | 0.6639 |
| 3 | rs11918634 | 7683434 | A | G | 0.4311 | 0.419 | 1.051 | 0.1472 | 0.7874 | 1.402 | 0.737 |
| 3 | rs9883258 | 7419156 | G | A | 0.4912 | 0.4965 | 0.979 | 0.1454 | 0.7361 | 1.302 | 0.8838 |
| 3 | rs342026 | 6908535 | A | G | 0.4894 | 0.4894 | 0.9999 | 0.1455 | 0.7518 | 1.33 | 0.9992 |

Abbreviations: CHR, chromosome; SNP, single nucleotide polymorphism; BP, base position; A1/A2, minor allele/major allele; F_P, frequency of minor allele in patients of poor response; F_G, frequency of minor allele in patients of good response; L95/U95, 95% Confidence interval; OR, odds ratio; SE, standard error.

**Supplementary Table 2. Association results of GRM7 SNPs with quetiapine treatment response**

| CHR | SNP | BP | A1 | A2 | F_P | F_G | OR | SE | L95 | U95 | P |
| --- | --- | --- | --- | --- | --- | --- | --- | --- | --- | --- | --- |
| 3 | rs78137319 | 7348332 | A | G | 0.03505 | 0.01163 | 3.087 | 0.5675 | 1.015 | 9.389 | 0.03685 |
| 3 | rs9883258 | 7419156 | G | A | 0.4743 | 0.5291 | 0.8031 | 0.145 | 0.6044 | 1.067 | 0.1303 |
| 3 | rs9882058 | 6937327 | C | T | 0.01168 | 0.02616 | 0.44 | 0.5625 | 0.1461 | 1.325 | 0.134 |
| 3 | rs13321431 | 7001961 | T | G | 0.4766 | 0.5203 | 0.8395 | 0.145 | 0.6319 | 1.115 | 0.2273 |
| 3 | rs1154370 | 7062465 | G | T | 0.493 | 0.4593 | 1.145 | 0.1451 | 0.8613 | 1.521 | 0.3517 |
| 3 | rs480409 | 7010081 | C | T | 0.3832 | 0.4128 | 0.8837 | 0.1479 | 0.6613 | 1.181 | 0.4031 |
| 3 | rs74668910 | 7068619 | T | G | 0.002336 | 0.005814 | 0.4005 | 1.227 | 0.03616 | 4.435 | 0.4402 |
| 3 | rs6778030 | 7153652 | G | A | 0.4813 | 0.4535 | 1.118 | 0.1452 | 0.8413 | 1.486 | 0.4414 |
| 3 | rs342026 | 6908535 | A | G | 0.5 | 0.4767 | 1.098 | 0.1449 | 0.8262 | 1.458 | 0.5206 |
| 3 | rs670764 | 7026341 | G | A | 0.4159 | 0.436 | 0.9209 | 0.1464 | 0.6911 | 1.227 | 0.5733 |
| 3 | rs7623046 | 7510856 | A | G | 0.486 | 0.5058 | 0.9237 | 0.1449 | 0.6954 | 1.227 | 0.5838 |
| 3 | rs1504047 | 7748843 | T | G | 0.3248 | 0.3081 | 1.08 | 0.1559 | 0.7957 | 1.466 | 0.6218 |
| 3 | rs17047886 | 7780116 | C | T | 0.01168 | 0.008721 | 1.344 | 0.7339 | 0.3188 | 5.662 | 0.6863 |
| 3 | rs779746 | 7578531 | T | C | 0.472 | 0.4855 | 0.9473 | 0.145 | 0.713 | 1.259 | 0.7089 |
| 3 | rs11918634 | 7683434 | A | G | 0.4206 | 0.4302 | 0.9612 | 0.1464 | 0.7214 | 1.281 | 0.787 |
| 3 | rs7374553 | 7528503 | G | A | 0.5023 | 0.4942 | 1.033 | 0.1448 | 0.7778 | 1.372 | 0.8219 |
| 3 | rs712767 | 7443688 | C | T | 0.4813 | 0.4884 | 0.9721 | 0.1449 | 0.7318 | 1.291 | 0.8452 |
| 3 | rs2291867 | 7340164 | G | A | 0.4953 | 0.4884 | 1.028 | 0.1448 | 0.7741 | 1.366 | 0.8476 |
| 3 | rs3749380 | 6903297 | T | C | 0.2827 | 0.2849 | 0.9894 | 0.1606 | 0.7222 | 1.355 | 0.9469 |

Abbreviations: CHR, chromosome; SNP, single nucleotide polymorphism; BP, base position; A1/A2, minor allele/major allele; F_P, frequency of minor allele in patients of poor response; F_G, frequency of minor allele in patients of good response; L95/U95, 95% Confidence interval; OR, odds ratio; SE, standard error.

**Supplementary Table 3. Association results of GRM7 SNPs with risperidone treatment response**

| CHR | SNP | BP | A1 | A2 | F_P | F_G | OR | SE | L95 | U95 | P |
| --- | --- | --- | --- | --- | --- | --- | --- | --- | --- | --- | --- |
| 3 | rs1154370 | 7062465 | G | T | 0.5036 | 0.4023 | 1.507 | 0.151 | 1.121 | 2.027 | 0.006439 |
| 3 | rs670764 | 7026341 | G | A | 0.457 | 0.3947 | 1.29 | 0.1515 | 0.9589 | 1.737 | 0.09209 |
| 3 | rs78137319 | 7348332 | A | G | 0.01075 | 0.02632 | 0.4022 | 0.5614 | 0.1338 | 1.209 | 0.09369 |
| 3 | rs342026 | 6908535 | A | G | 0.5143 | 0.4549 | 1.269 | 0.1495 | 0.9469 | 1.701 | 0.1105 |
| 3 | rs9883258 | 7419156 | A | G | 0.5179 | 0.4586 | 1.268 | 0.1494 | 0.9462 | 1.699 | 0.1116 |
| 3 | rs480409 | 7010081 | C | T | 0.4337 | 0.3797 | 1.251 | 0.1525 | 0.9278 | 1.687 | 0.1415 |
| 3 | rs7374553 | 7528503 | G | A | 0.4857 | 0.4323 | 1.24 | 0.15 | 0.9241 | 1.664 | 0.1514 |
| 3 | rs712767 | 7443688 | C | T | 0.4695 | 0.4173 | 1.236 | 0.1505 | 0.9202 | 1.66 | 0.159 |
| 3 | rs7623046 | 7510856 | A | G | 0.4821 | 0.5301 | 0.8252 | 0.1492 | 0.6159 | 1.106 | 0.1976 |
| 3 | rs17047886 | 7780116 | C | T | 0.008961 | 0.0188 | 0.472 | 0.6369 | 0.1355 | 1.645 | 0.2279 |
| 3 | rs3749380 | 6903297 | T | C | 0.2527 | 0.2857 | 0.8453 | 0.1671 | 0.6093 | 1.173 | 0.3142 |
| 3 | rs74668910 | 7068619 | T | G | 0.01434 | 0.007519 | 1.92 | 0.7941 | 0.4049 | 9.104 | 0.4033 |
| 3 | rs1504047 | 7748843 | T | G | 0.3011 | 0.3233 | 0.9016 | 0.1603 | 0.6585 | 1.234 | 0.5182 |
| 3 | rs6778030 | 7153652 | G | A | 0.4821 | 0.4662 | 1.066 | 0.1493 | 0.7955 | 1.428 | 0.6689 |
| 3 | rs9882058 | 6937327 | C | T | 0.01254 | 0.01504 | 0.8321 | 0.6313 | 0.2415 | 2.868 | 0.7707 |
| 3 | rs2291867 | 7340164 | G | A | 0.4946 | 0.5038 | 0.9641 | 0.149 | 0.7199 | 1.291 | 0.8063 |
| 3 | rs11918634 | 7683434 | A | G | 0.3781 | 0.3835 | 0.9777 | 0.1534 | 0.7238 | 1.321 | 0.883 |
| 3 | rs13321431 | 7001961 | T | G | 0.4695 | 0.4662 | 1.014 | 0.1493 | 0.7564 | 1.358 | 0.9278 |
| 3 | rs779746 | 7578531 | C | T | 0.4642 | 0.4624 | 1.007 | 0.1494 | 0.7514 | 1.35 | 0.9624 |

Abbreviations: CHR, chromosome; SNP, single nucleotide polymorphism; BP, base position; A1/A2, minor allele/major allele; F_P, frequency of minor allele in patients of poor response; F_G, frequency of minor allele in patients of good response; L95/U95, 95% Confidence interval; OR, odds ratio; SE, standard error.

**Supplementary Table 4. Association results of GRM7 SNPs with haloperidol treatment response**

| CHR | SNP | BP | A1 | A2 | F_P | F_G | OR | SE | L95 | U95 | P |
| --- | --- | --- | --- | --- | --- | --- | --- | --- | --- | --- | --- |
| 3 | rs1154370 | 7062465 | G | T | 0.5531 | 0.4177 | 1.725 | 0.2096 | 1.144 | 2.602 | 0.00903 |
| 3 | rs712767 | 7443688 | C | T | 0.4558 | 0.538 | 0.7192 | 0.2081 | 0.4783 | 1.081 | 0.1127 |
| 3 | rs480409 | 7010081 | C | T | 0.3938 | 0.3165 | 1.403 | 0.2186 | 0.9142 | 2.154 | 0.1206 |
| 3 | rs9882058 | 6937327 | C | T | 0.004425 | 0.01899 | 0.2296 | 1.159 | 0.02367 | 2.228 | 0.1666 |
| 3 | rs6778030 | 7153652 | G | A | 0.5 | 0.4304 | 1.324 | 0.2086 | 0.8794 | 1.992 | 0.1787 |
| 3 | rs74668910 | 7068619 | T | G | 0.02212 | 0.006329 | 3.552 | 1.1 | 0.411 | 30.7 | 0.2194 |
| 3 | rs13321431 | 7001961 | T | G | 0.4646 | 0.5253 | 0.7841 | 0.2078 | 0.5218 | 1.178 | 0.2415 |
| 3 | rs342026 | 6908535 | A | G | 0.4602 | 0.519 | 0.7901 | 0.2078 | 0.5258 | 1.187 | 0.2565 |
| 3 | rs7374553 | 7528503 | G | A | 0.4956 | 0.443 | 1.235 | 0.2082 | 0.8212 | 1.857 | 0.3103 |
| 3 | rs9883258 | 7419156 | G | A | 0.4602 | 0.5127 | 0.8104 | 0.2077 | 0.5394 | 1.218 | 0.3111 |
| 3 | rs78137319 | 7348332 | A | G | 0.0177 | 0.03165 | 0.5514 | 0.679 | 0.1457 | 2.086 | 0.374 |
| 3 | rs17047886 | 7780116 | C | T | 0.0177 | 0.03165 | 0.5514 | 0.679 | 0.1457 | 2.086 | 0.374 |
| 3 | rs1504047 | 7748843 | T | G | 0.2389 | 0.2785 | 0.8134 | 0.2363 | 0.5119 | 1.293 | 0.3818 |
| 3 | rs11918634 | 7683434 | A | G | 0.4558 | 0.4114 | 1.198 | 0.2097 | 0.7943 | 1.807 | 0.3885 |
| 3 | rs2291867 | 7340164 | A | G | 0.4912 | 0.4494 | 1.183 | 0.208 | 0.7867 | 1.778 | 0.4197 |
| 3 | rs7623046 | 7510856 | A | G | 0.4558 | 0.4304 | 1.108 | 0.2089 | 0.7359 | 1.669 | 0.6225 |
| 3 | rs670764 | 7026341 | G | A | 0.4292 | 0.4051 | 1.104 | 0.2105 | 0.731 | 1.669 | 0.6371 |
| 3 | rs779746 | 7578531 | C | T | 0.4558 | 0.443 | 1.053 | 0.2085 | 0.6995 | 1.584 | 0.8054 |
| 3 | rs3749380 | 6903297 | T | C | 0.292 | 0.2911 | 1.004 | 0.2282 | 0.6422 | 1.571 | 0.9848 |

Abbreviations: CHR, chromosome; SNP, single nucleotide polymorphism; BP, base position; A1/A2, minor allele/major allele; F_P, frequency of minor allele in patients of poor response; F_G, frequency of minor allele in patients of good response; L95/U95, 95% Confidence interval; OR, odds ratio; SE, standard error.

**Supplementary Table 5. Association results of GRM7 SNPs with perphenazine treatment response**

| CHR | SNP | BP | A1 | A2 | F_P | F_G | OR | SE | L95 | U95 | P |
| --- | --- | --- | --- | --- | --- | --- | --- | --- | --- | --- | --- |
| 3 | rs779746 | 7578531 | T | C | 0.433 | 0.5556 | 0.611 | 0.2078 | 0.4066 | 0.9182 | 0.01745 |
| 3 | rs9883258 | 7419156 | G | A | 0.442 | 0.5432 | 0.666 | 0.2073 | 0.4436 | 0.9999 | 0.0495 |
| 3 | rs712767 | 7443688 | C | T | 0.5 | 0.4012 | 1.492 | 0.2087 | 0.9913 | 2.246 | 0.05462 |
| 3 | rs1154370 | 7062465 | G | T | 0.4509 | 0.5309 | 0.7257 | 0.2069 | 0.4837 | 1.089 | 0.1208 |
| 3 | rs6778030 | 7153652 | G | A | 0.5 | 0.4321 | 1.314 | 0.2074 | 0.8753 | 1.973 | 0.1872 |
| 3 | rs480409 | 7010081 | C | T | 0.3795 | 0.4444 | 0.7644 | 0.2097 | 0.5068 | 1.153 | 0.1996 |
| 3 | rs342026 | 6908535 | G | A | 0.4464 | 0.5123 | 0.7676 | 0.2068 | 0.5118 | 1.151 | 0.2006 |
| 3 | rs3749380 | 6903297 | T | C | 0.3036 | 0.2531 | 1.286 | 0.2319 | 0.8166 | 2.027 | 0.2769 |
| 3 | rs9882058 | 6937327 | C | T | 0.004464 | 0.0125 | 0.3543 | 1.229 | 0.03185 | 3.941 | 0.3779 |
| 3 | rs74668910 | 7068619 | T | G | 0.004464 | 0 | NA | NA | NA | NA | 0.3945 |
| 3 | rs7374553 | 7528503 | G | A | 0.4866 | 0.4506 | 1.156 | 0.2069 | 0.7704 | 1.733 | 0.4845 |
| 3 | rs13321431 | 7001961 | G | T | 0.4821 | 0.5062 | 0.9083 | 0.2063 | 0.6062 | 1.361 | 0.6412 |
| 3 | rs2291867 | 7340164 | A | G | 0.4955 | 0.4753 | 1.084 | 0.2064 | 0.7235 | 1.625 | 0.6948 |
| 3 | rs11918634 | 7683434 | A | G | 0.4643 | 0.4444 | 1.083 | 0.2072 | 0.7217 | 1.626 | 0.6993 |
| 3 | rs78137319 | 7348332 | A | G | 0.008929 | 0.006173 | 1.45 | 1.229 | 0.1304 | 16.13 | 0.7609 |
| 3 | rs1504047 | 7748843 | T | G | 0.3259 | 0.3148 | 1.052 | 0.2212 | 0.682 | 1.623 | 0.8181 |
| 3 | rs670764 | 7026341 | G | A | 0.442 | 0.4321 | 1.041 | 0.208 | 0.6924 | 1.565 | 0.8471 |
| 3 | rs7623046 | 7510856 | G | A | 0.4866 | 0.4938 | 0.9715 | 0.2063 | 0.6484 | 1.456 | 0.8886 |
| 3 | rs17047886 | 7780116 | C | T | 0.01339 | 0.01235 | 1.086 | 0.9188 | 0.1794 | 6.575 | 0.9285 |

Abbreviations: CHR, chromosome; SNP, single nucleotide polymorphism; BP, base position; A1/A2, minor allele/major allele; F_P, frequency of minor allele in patients of poor response; F_G, frequency of minor allele in patients of good response; L95/U95, 95% Confidence interval; OR, odds ratio; SE, standard error.

**Supplementary Table 6. Association results of GRM7 SNPs with ziprasidone treatment response**

| CHR | SNP | BP | A1 | A2 | F_P | F_G | OR | SE | L95 | U95 | P |
| --- | --- | --- | --- | --- | --- | --- | --- | --- | --- | --- | --- |
| 3 | rs6778030 | 7153652 | G | A | 0.445 | 0.4975 | 0.8098 | 0.1405 | 0.6149 | 1.067 | 0.1331 |
| 3 | rs670764 | 7026341 | G | A | 0.4282 | 0.3894 | 1.174 | 0.1426 | 0.8879 | 1.553 | 0.2601 |
| 3 | rs9882058 | 6937327 | C | T | 0.01914 | 0.01005 | 1.922 | 0.6164 | 0.5742 | 6.433 | 0.281 |
| 3 | rs712767 | 7443688 | C | T | 0.4426 | 0.4774 | 0.8692 | 0.1406 | 0.6599 | 1.145 | 0.3187 |
| 3 | rs7623046 | 7510856 | A | G | 0.4928 | 0.4673 | 1.108 | 0.1402 | 0.8414 | 1.458 | 0.4664 |
| 3 | rs1154370 | 7062465 | G | T | 0.488 | 0.4648 | 1.098 | 0.1403 | 0.8337 | 1.445 | 0.5069 |
| 3 | rs9883258 | 7419156 | G | A | 0.4569 | 0.4799 | 0.9119 | 0.1404 | 0.6925 | 1.201 | 0.5111 |
| 3 | rs779746 | 7578531 | C | T | 0.4569 | 0.4799 | 0.9119 | 0.1404 | 0.6925 | 1.201 | 0.5111 |
| 3 | rs78137319 | 7348332 | A | G | 0.01196 | 0.007538 | 1.594 | 0.7337 | 0.3784 | 6.714 | 0.5214 |
| 3 | rs74668910 | 7068619 | T | G | 0.004785 | 0.007538 | 0.633 | 0.9156 | 0.1052 | 3.808 | 0.6145 |
| 3 | rs3749380 | 6903297 | T | C | 0.2799 | 0.2915 | 0.945 | 0.155 | 0.6973 | 1.28 | 0.7149 |
| 3 | rs480409 | 7010081 | C | T | 0.3565 | 0.3668 | 0.9561 | 0.1458 | 0.7185 | 1.272 | 0.7578 |
| 3 | rs1504047 | 7748843 | T | G | 0.2967 | 0.3065 | 0.9542 | 0.1526 | 0.7075 | 1.287 | 0.7585 |
| 3 | rs342026 | 6908535 | G | A | 0.4928 | 0.4824 | 1.043 | 0.1401 | 0.7922 | 1.372 | 0.7662 |
| 3 | rs7374553 | 7528503 | G | A | 0.4641 | 0.4548 | 1.038 | 0.1405 | 0.7883 | 1.368 | 0.789 |
| 3 | rs17047886 | 7780116 | C | T | 0.01442 | 0.01256 | 1.15 | 0.6096 | 0.3482 | 3.799 | 0.8183 |
| 3 | rs13321431 | 7001961 | T | G | 0.4952 | 0.5025 | 0.9712 | 0.1401 | 0.7381 | 1.278 | 0.8349 |
| 3 | rs2291867 | 7340164 | A | G | 0.4856 | 0.4899 | 0.9829 | 0.1401 | 0.7469 | 1.294 | 0.9022 |
| 3 | rs11918634 | 7683434 | A | G | 0.4043 | 0.407 | 0.9887 | 0.1426 | 0.7476 | 1.308 | 0.9367 |

Abbreviations: CHR, chromosome; SNP, single nucleotide polymorphism; BP, base position; A1/A2, minor allele/major allele; F_P, frequency of minor allele in patients of poor response; F_G, frequency of minor allele in patients of good response; L95/U95, 95% Confidence interval; OR, odds ratio; SE, standard error.

**Supplementary Table 7. Association results of GRM7 SNPs with aripiprazole treatment response**

| CHR | SNP | BP | A1 | A2 | F_P | F_G | OR | SE | L95 | U95 | P |
| --- | --- | --- | --- | --- | --- | --- | --- | --- | --- | --- | --- |
| 3 | rs480409 | 7010081 | C | T | 0.434 | 0.3676 | 1.319 | 0.1457 | 0.9914 | 1.755 | 0.05708 |
| 3 | rs9883258 | 7419156 | G | A | 0.4599 | 0.527 | 0.7642 | 0.1426 | 0.5778 | 1.011 | 0.05913 |
| 3 | rs342026 | 6908535 | G | A | 0.4575 | 0.5189 | 0.782 | 0.1426 | 0.5913 | 1.034 | 0.08435 |
| 3 | rs9882058 | 6937327 | C | T | 0.009434 | 0.02432 | 0.382 | 0.6052 | 0.1167 | 1.251 | 0.09908 |
| 3 | rs3749380 | 6903297 | T | C | 0.3042 | 0.2541 | 1.284 | 0.1594 | 0.9395 | 1.755 | 0.1165 |
| 3 | rs2291867 | 7340164 | G | A | 0.5165 | 0.4622 | 1.243 | 0.1425 | 0.9402 | 1.644 | 0.1265 |
| 3 | rs7623046 | 7510856 | G | A | 0.4906 | 0.4405 | 1.223 | 0.1428 | 0.9243 | 1.618 | 0.1587 |
| 3 | rs78137319 | 7348332 | A | G | 0.009434 | 0.005405 | 1.752 | 0.869 | 0.3191 | 9.623 | 0.5132 |
| 3 | rs7374553 | 7528503 | G | A | 0.4811 | 0.4595 | 1.091 | 0.1426 | 0.8249 | 1.443 | 0.5417 |
| 3 | rs17047886 | 7780116 | C | T | 0.01887 | 0.01351 | 1.404 | 0.5746 | 0.4552 | 4.329 | 0.5531 |
| 3 | rs712767 | 7443688 | C | T | 0.4599 | 0.4405 | 1.081 | 0.143 | 0.817 | 1.431 | 0.5843 |
| 3 | rs6778030 | 7153652 | G | A | 0.4835 | 0.4649 | 1.078 | 0.1425 | 0.815 | 1.425 | 0.6001 |
| 3 | rs1504047 | 7748843 | T | G | 0.2877 | 0.3027 | 0.9306 | 0.1559 | 0.6855 | 1.263 | 0.6445 |
| 3 | rs779746 | 7578531 | C | T | 0.4717 | 0.4595 | 1.05 | 0.1426 | 0.7942 | 1.389 | 0.7302 |
| 3 | rs11918634 | 7683434 | A | G | 0.4363 | 0.4243 | 1.05 | 0.1437 | 0.7924 | 1.392 | 0.7335 |
| 3 | rs74668910 | 7068619 | T | G | 0.01887 | 0.02162 | 0.8702 | 0.5051 | 0.3233 | 2.342 | 0.783 |
| 3 | rs670764 | 7026341 | G | A | 0.4222 | 0.4135 | 1.036 | 0.1443 | 0.781 | 1.375 | 0.8052 |
| 3 | rs1154370 | 7062465 | G | T | 0.4623 | 0.4703 | 0.9683 | 0.1426 | 0.7322 | 1.281 | 0.8215 |
| 3 | rs13321431 | 7001961 | T | G | 0.4906 | 0.4919 | 0.9947 | 0.1423 | 0.7526 | 1.315 | 0.9703 |

Abbreviations: CHR, chromosome; SNP, single nucleotide polymorphism; BP, base position; A1/A2, minor allele/major allele; F_P, frequency of minor allele in patients of poor response; F_G, frequency of minor allele in patients of good response; L95/U95, 95% Confidence interval; OR, odds ratio; SE, standard error.

**Supplementary Figure 1. the allele frequencies of rs1516569 across populations (**[**http://grch37.ensembl.org**](http://grch37.ensembl.org)**)**


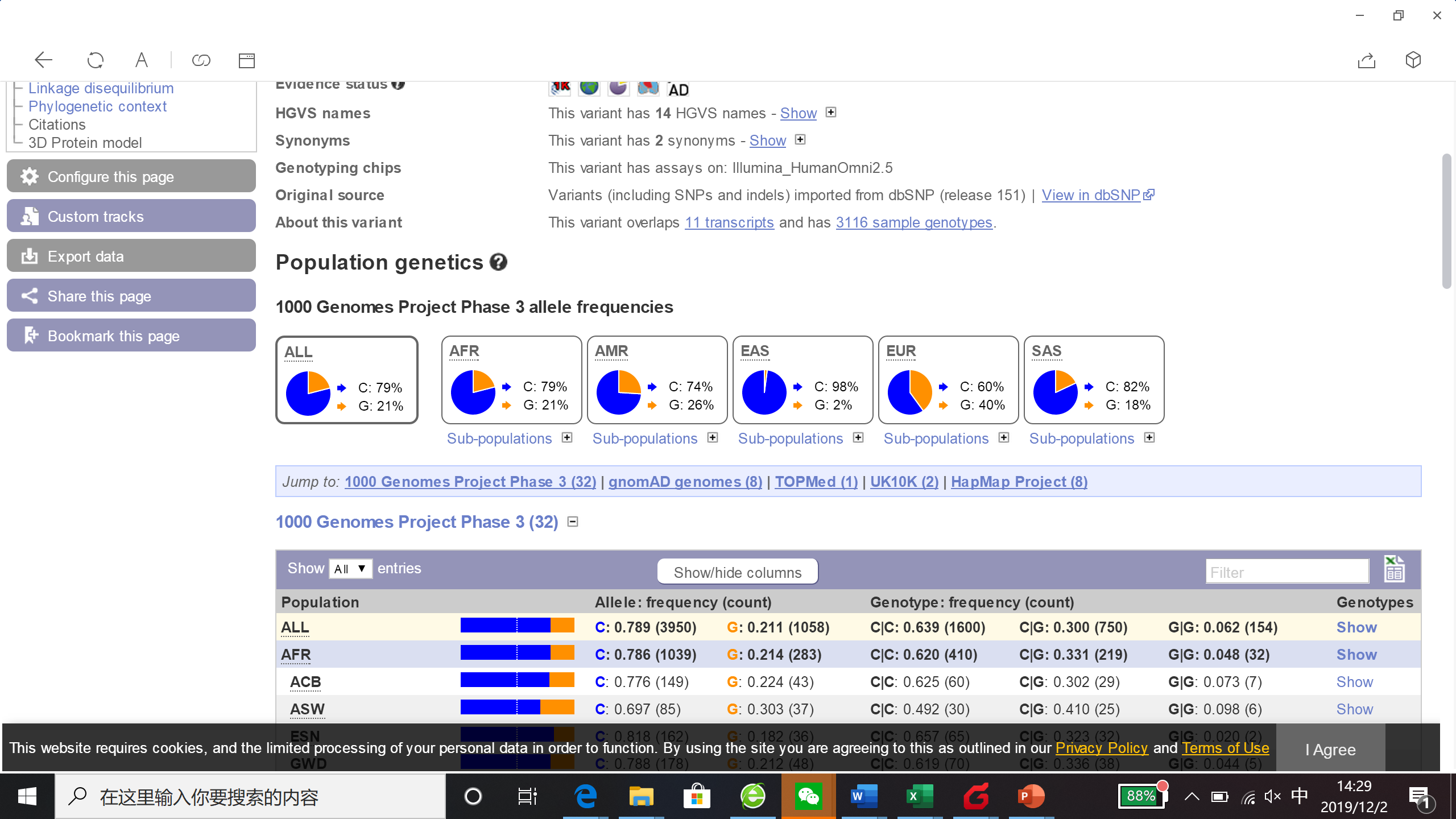


**Supplementary Figure 2. the allele frequencies of rs9883258 across populations (**[**http://grch37.ensembl.org**](http://grch37.ensembl.org)**)**


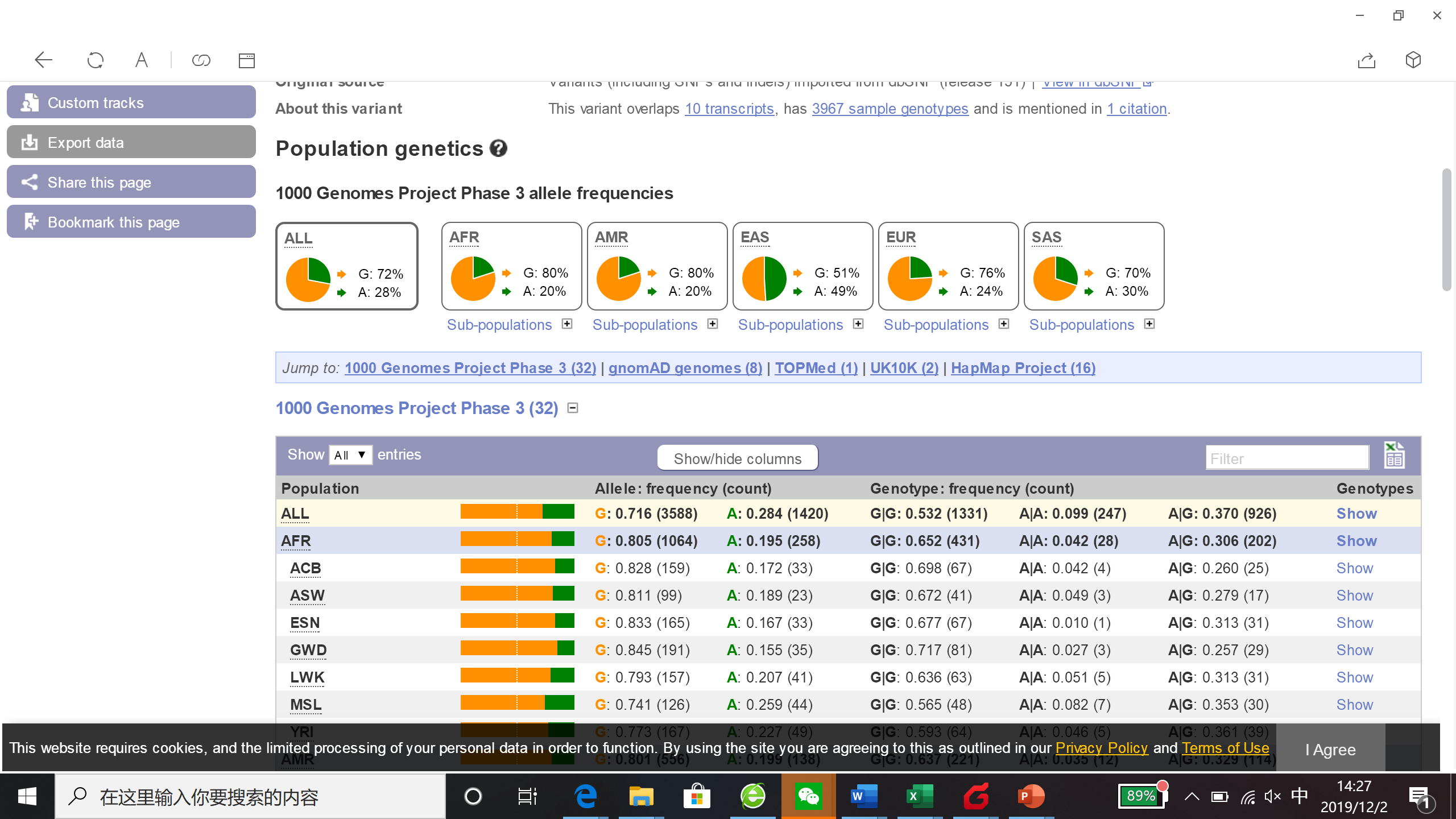

Supplement: Supplementary file 1 — Supplementary information [file 41398_2020_763_MOESM1_ESM.docx]
